# The role of SARS-COV-2 infection in promoting abnormal immune response and sepsis: A comparison between SARS-COV-2-related sepsis and sepsis from other causes

**DOI:** 10.1016/j.imj.2023.04.006

**Published:** 2023-04-28

**Authors:** Andrea Piccioni, Laura Franza, Federico Rosa, Marcello Candelli, Marcello Covino, Michela Ferrara, Gianpietro Volonnino, Giuseppe Bertozzi, Maria Vittoria Zamponi, Aniello Maiese, Gabriele Savioli, Francesco Franceschi, Raffaele La Russa

**Affiliations:** a Department of Emergency Medicine, Fondazione Policlinico Universitario A. Gemelli IRCCS, 1-00168 Rome, Italy; b Università Cattolica del Sacro Cuore, 1-00168 Rome, Italy; c Department of Anatomical, Histological, Forensic and Orthopedic Sciences, Sapienza University of Rome, 00186 Rome, Italy; d Department of Clinical and Experimental Medicine, Institute of Legal Medicine, University of Foggia, 71100 Foggia, Italy; e Department of Surgical, Medical, and Molecular Pathology and Critical Care Medicine, University of Pisa, via Roma 55, 56126 Pisa, Italy; f Emergency Department, IRCCS Policlinico San Matteo, 27100 Pavia, Italy; g Department of Clinical-Surgical, Diagnostic and Pediatric Sciences, University of Pavia, 27100 Pavia, Italy

**Keywords:** SARS-COV-2, Cytokine storm, SARS-CoV-2, Septic shock, Sepsis

## Abstract

**Background:**

COVID-19 caused by SARS-CoV-2 virus is characterized by respiratory compromise and immune system involvement, even leading to serious disorders, such as cytokine storm.

**Methods:**

We then conducted a literature review on the topic of sepsis and covid-19, and in parallel conducted an experimental study on the histological finding of patients who died from SARS-Covid 19 infection and a control group.

**Results:**

Sepsis associated with covid-19 infection has some similarities and differences from that from other causes.

**Conclusion:**

In this paper the complex interplay between the 2 disorders was discussed, focusing on the similarities and on the effect that one could have on the other. A preliminary experimental section that demonstrates the multisystemic involvement in subjects who die from SARS-CoV-2 is also proposed.

## Graphical abstract

**Figure:**
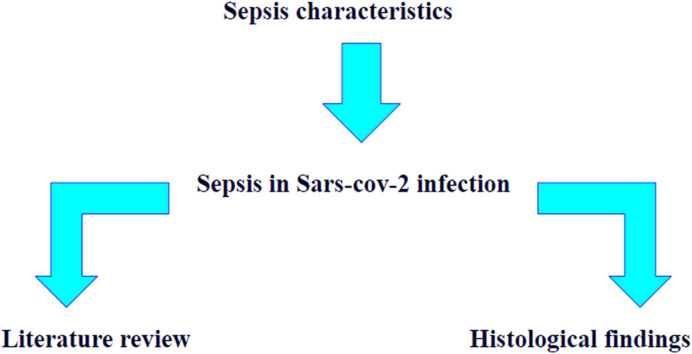

## 1 Introduction

SARS-CoV-2 has completely changed the global health perspective. In the last 2 years, needs and resources changed drastically in the face of the rise of SARS-CoV-2, which has become the number one concern for the health system across the world.

Other diseases have thus been treated differently, in terms of therapy timing and overall approach [1], [2], [3]. In particular, in the context of emergency care, physicians had to find ways to treat patients as fast as possible, while still preserving their health [4]. The impact of SARS-CoV-2 was minimal in treating diseases for which clear pathways of treatment were already known and standardized, for instance, stroke [5] and acute myocardial infarction [6], even though a reduction in the number of patients presenting to the emergency department was registered [7]. On the other hand, patients presenting with serious diseases, needing fast medical attention and therapy, but without standardized protocols, might have experienced suboptimal care [8].

An example of such a situation is sepsis. Sepsis is a complicated disease, characterized by an abnormal response to infection, which can lead to shock and, if not treated adequately, death [9]. Interestingly, SARS-CoV-2 infection can also lead to a similar condition, through a condition known as a cytokine storm [10]. It is now agreed that patients experiencing severe SARS-CoV-2 infection are experiencing it because of the immune response the virus elicited, rather than direct viral damage [11].

According to the Surviving Sepsis Campaign, a timely diagnosis must be promoted [12,13], although septic patients presenting to the emergency department during the current pandemic were difficult to identify, also because, in some cases, it was SARS-CoV-2 itself presenting as septic shock, severely impacting prognosis of those kind of patients [14], [15], [16].

In the present manuscript, the complex interplay between the 2 disorders is addressed, through a narrative review of the evidence present in literature. A preliminary experimental section that demonstrates the multisystemic involvement in subjects who died from SARS-CoV-2 is also presented.

The aim of our work is to better define the relationship between sepsis and Sars-cov-2 infection. For this reason, in a first section we will analyze the most important studies that have dealt with this subject, while in a second section we will present our histological results obtained in patients who died of covid-19 infection.

## 2 Materials and methods

### 2.1 Literature review

A narrative review was performed using MEDLINE and Google Scholar from January 2020 up to 28th May 2020, to identify the coagulative state in patients with SARS-COV-2. We included the following search terms: “SARS-COV-2” and “SARS-CoV-2” in combination with “cytokine storm”, “sepsis”, and “septic shock”. The reference lists of all studies included were manually searched to identify any other study that might merit inclusion. We excluded articles in non-English-language, or not relevant topics to the specific focus of this review.

Finally, among the 786 papers identified, 160 articles were selected after the title and abstract examination, and the removal of duplicates. Finally, only 82 articles were analyzed because they focused on our review guidelines.

The data processing complied with the general authorization for scientific research purposes granted by the Italian Data Protection Authority (1 March 2012 as published in Italy's Official Journal no. 72 dated 26 March 2012) since the data do not entail any significant personalized impact on the data subjects. Approval by an institutional and/or licensing committee was not required since experimental protocols were not applied in the study (This statement is appropriate because the manuscript includes data from a human sample but experimental protocols were not applied, so it was not necessary the approval by an institutional and/or licensing committee). Protocols and screening were conducted as suggested by the World Health Organization and in conformity with the ethical guidelines of the 1975 Declaration of Helsinki.

### 2.2 Experimental study

#### 2.2.1 Case selection

Ten subjects (average age 65 years) who died from SARS-Covid 19 infection, with a certain ante-mortem diagnosis of COVID-19, were selected for this experimental study. In all these cases the nasopharyngeal swab was positive for SARS-Covid 19 and the ante-mortem CT showed SARS-Covid-related viral pneumonia. The exclusion criterion was the presence of concomitant lung infections. Before the autopsy, swabs of the upper respiratory tract (nasopharynx and oropharynx) were collected, and all confirmed positivity for SARS-Covid 19. Furthermore, procalcitonin, a known marker of sepsis, was not measured ante-mortem in all subjects.

The Control Group included 5 subjects who died before 2018 with causes of death other than infection: one died from opioid overdose; one died from hanging; 2 subjects died after car accidents, and the last died from sudden cardiac death.

#### 2.2.2 Histology

**Fig. 1:**
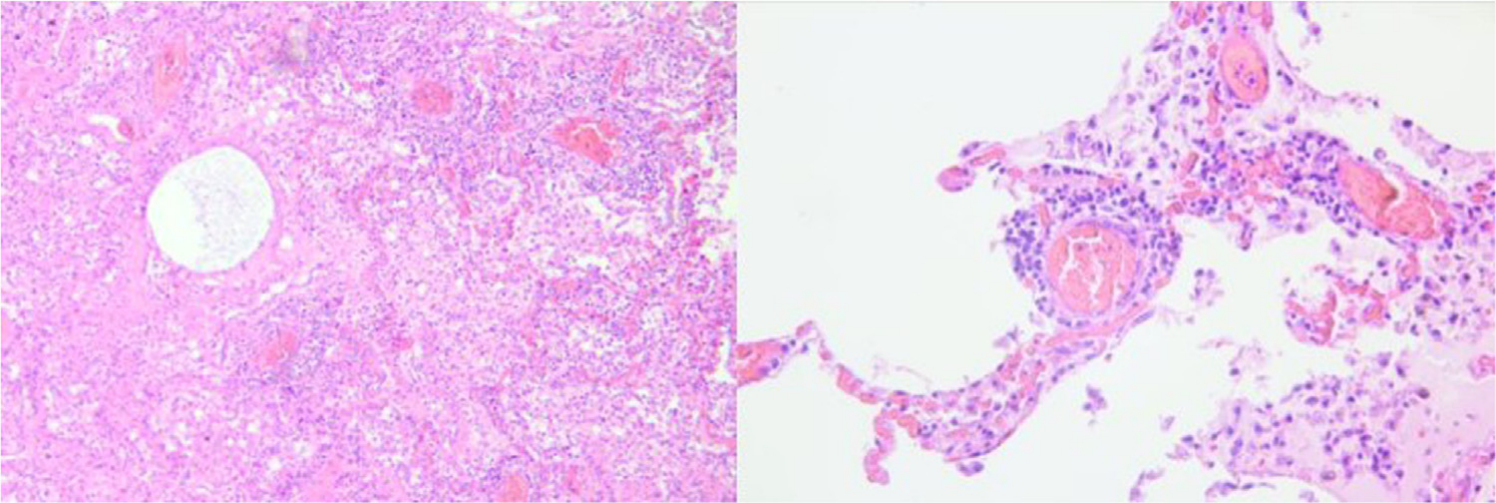
Histological findings (H&E, × 40, × 60): congestion, edema fluid focally, and perivascular lymphocytic cuffing (arrow black) and inflammatory cells within the septa.

Standard hematoxylin-eosin staining was performed for each sample. SARS-CoV-2 samples histologically showed diffuse alveolar damage (DAD), desquamation of hyperplastic pneumocytes, and presence of multinucleated cells and foamy macrophages were observed (Fig. 1). In addition, the pulmonary vessels showed vasculitic alterations and small arteries showed fibrin thrombi.

Immunohistochemistry (IHC) was then carried out on formalin-fixed paraffin-embedded tissue sections (4 µm), after being de-waxed and then rehydrated. These blocks were sectioned and stained on a benchmark XT system (Ventana) with an antibody directed against procalcitonin (clone 44d9, Novusbio). The antiprocalcitonin monoclonal antibody was diluted at 1:150. Antigen retrieval was carried out with an automated process, using a Benchmark XT for 32 minutes, at a temperature of 37 °C.

Semiquantitative analysis was performed with an optic microscope [17], grading the positive reaction as follows: 0 (−) not expressed; 1 (+) isolated and disseminated expression; 2 (++) expression in scattered foci; 3 (+++) expression in widespread foci; 4 (++++) widespread expression. The evaluations were carried out separately for each tissue, using a double-blind method between 2 observers. In cases of divergent scoring, a third observer decided the final score.

## 3 Results

### 3.1 Sepsis: an ever changing disease?

**Fig. 2:**
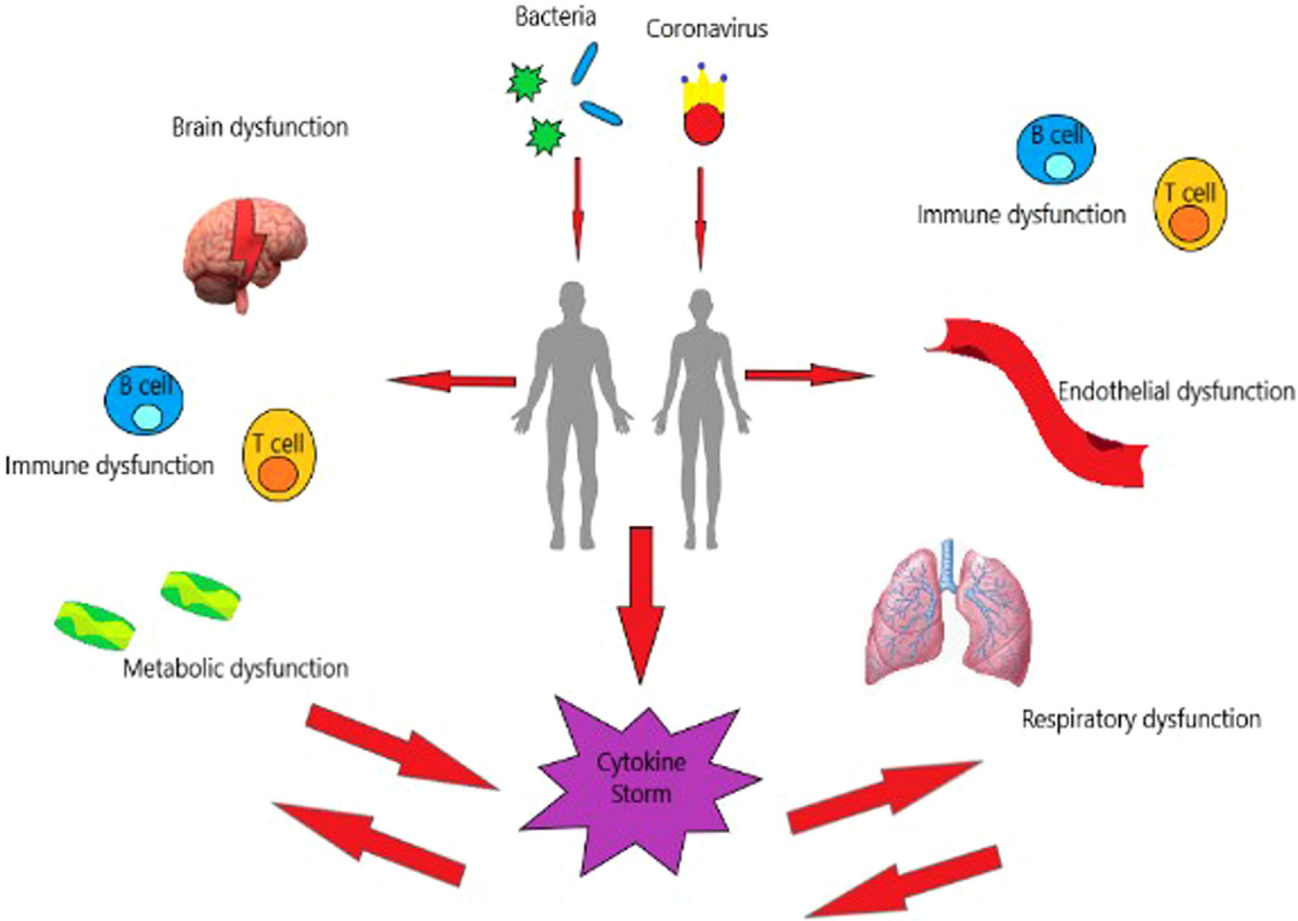
Sepsis and SARS-CoV-2 are both complicated diseases in which a variety of organs and systems are involved. In particular, in sepsis, brain dysfunction, and metabolic alterations are predominant, while in SARS-CoV-2 endothelial and lung dysfunction are common. Yet, other organs can be targeted as well and sometimes their characteristics can overlap. Interestingly in both conditions, a cytokine storm can take place and trigger at least in part the different dysfunctions.

While sepsis is an easy-to-grasp concept for the physician working in an acute care setting, its definition has changed throughout time (Fig. 2).

First definitions of sepsis focused mainly on the presence of 2 or more systemic inflammatory syndrome response criteria (SIRS), in the context of a known or strongly suspected infection, and patients were stratified based on the severity of their conditions [18,19]. Throughout the years the definition has changed time and time again, to try and correctly identify septic patients. The most recent consensus has agreed on defining sepsis as “the body's extreme response to an infection”. It is a life-threatening medical emergency. Sepsis takes place when an infection you already have, triggers a chain reaction throughout your body. Infections that lead to sepsis most often start in the lung, urinary tract, skin, or gastrointestinal tract [20].

The complexity [21], [22], [23], [24] of defining sepsis mirrors the difficulties in correctly diagnosing it. Several scores have been designed in the years to identify septic patients and stratify their overall risk of adverse outcomes. More specifically, sequential organ failure assessment (SOFA), quick SOFA (qSOFA), and SIRS criteria are the scores used to identify and classify septic patients [9,25].

The difficulties in diagnosis, combined with the necessity to treat the disease as soon as possible, partly account for the enormous burden on health systems all over the world.

It is estimated that sepsis is one of the main causes of in-hospital mortality in Western countries. Despite technical and cultural advances in understanding pathophysiology and ongoing research on the most appropriate treatment, sepsis still carries a high mortality, reaching over 20% [26]. Also, surviving patients affected by sepsis often require long-term therapies and rehabilitation. Patients are also susceptible to long-term sequelae, which represent an important burden on both patients and healthcare facilities [27].

### 3.2 The burden of SARS-COV-2 on global health

Toward the end of 2019, a new coronavirus, called SARS-CoV-2 [28], has been first reported in Wuhan City of China [29].

The disease it caused was named SARS-CoV-2 and from that moment until November 2021, more than 250 million people worldwide were infected, and over 5 million people died [30].

The disease has a wide variety of presentations and, while in most cases it is a flu-like disease or even asymptomatic, it can also determine a fast-progressing illness, leading to respiratory disease and death. The groups at a major risk of such evolution are elders or people who are immunocompromised [31,32]. In particular, in the literature it has been observed that the highest mortality rate among patients suffering from COVID-19, was among male patients and over 70 years of age. The same result is shown in patients with specific symptoms such as productive cough, in patients with multimorbidity and with pre-existing polypharmacy at the time of onset of infection [10.1007/s11739-021-02742-8].

Also, it is worth noting that while respiratory symptoms are the most common, also other organs and systems can be involved. For instance, neurological involvement, comprising headache, confusion, delirium, anosmia or hyposmia, dysgeusia or ageusia, altered mental status, ataxia, and seizures [33]. Other patients have experienced gastrointestinal disorders, or cardiovascular, putting enormous stress on health providers. Another aspect that is important to take into consideration is that some patients who were infected with SARS-CoV-2 also developed long-term symptoms, a syndrome renamed long-Covid [34]. The symptoms patients can experience long-term vary widely, from respiratory to hematologic, but the most prevalent symptom is chronic fatigue [35]. Overall, it has been estimated that these patients will require intense rehabilitation and long-term care, once again putting an incredible burden on already underpressure health systems [36,37].

It appeared clear at the beginning of the pandemic that it would be necessary to put in place measures to contain the virus, ranging from limitations to certain activities to complete lockdown in many countries [38]. Yet even these measures were not completely effective and led to a crisis in mental health, particularly among adolescents [39].

Patients with other diseases were also impacted by the pandemic, with a delay in care which proved to be even fatal in some circumstances [40]. The pandemic has also caused a significant economic crisis, forever changing the lives of thousands of people, since it led to the largest recession since the end of World War II [41], and has had a major impact on the mental health of all citizens, not just health professionals [42]. The different lockdowns, which at the beginning were the only measure against the spread of the virus, damaged the economy, leaving some people in dire conditions, which further impacted their health [43]. The impact of the disease has changed after several types of SARS-CoV-2 vaccines have been approved. According to the report of the Istituto Superiore di Sanità (ISS) in Italy, vaccinated people have a 78% lower risk of contracting SARS-CoV-2 infection, 94% of being hospitalized, 96% of being assisted in intensive care, and 97% to die [44].

Since the beginning of the pandemic, different researchers began to look for an effective vaccine against this disease and over 140 different types of vaccines are being studied. At the moment 4 are currently approved in Italy [45].

Of these 4 vaccines, 2 are mRNA based: Pfizer mRNABNT162b2 (Comirnaty) and SARS-COV-2 Vaccine Moderna mRNA-1273 (Spikevax), while 2 are viral vectors: Vaxzevria e SARS-COV-2 Vaccine Janssen [46].

In Italy in November 2021, over 83% of the population completed the vaccination cycle [47]. Yet, while vaccinations are proceeding steadily, it is, unfortunately, becoming more and more clear that even small parts of the population who are not vaccinated pose a great risk for the rest of the world [48].

### 3.3 Immune pathways in SARS-COV-2

From the beginning of the pandemic, it appeared clear that SARS-CoV-2 was not simply a pulmonary disease. The first accounts of altered immunity during the infection appeared as soon as February and, already in March, authors had identified the cytokine storm as a possible leading actor in determining Covid's morbidity burden [49].

SARS-CoV-2 infection appears to be capable of determining abnormal immune responses in some people. This was first suspected when physicians observed that a group of patients experienced a worsening of their conditions around day 10 of the infection [50]: patients who seemed to be improving suddenly developed worsening symptoms, even though in some cases they tested negative for an active infection [51].

It soon became obvious that the wide variety of symptoms patients were experiencing was not a consequence of direct infection, but rather to the immune response to the virus, similarly to what happens during sepsis [52]. The unfavorable outcome can indeed be predicted by laboratory alterations such as elevated levels of inflammatory markers such as procalcitonin, interleukin (IL)-6, and reduction in the number of leukocytes [53,54], which are also sepsis markers.

The first stages of the immune response to SARS-CoV-2 are characterized by the activation of the innate immune system, with the involvement of the interferon regulatory system and the nuclear factor kappa B (NF-κB). The evolution of this stage determines further progression: overactivation of macrophages is determined in these first moments of the infection and is one of the main actors in triggering a cytokine storm [55].

A cytokine storm is a known entity in the context of several immune disorders [31] and is characterized by a vicious circle, in which inflammatory cytokines, particularly IL-6, IL-1, and tumor necrosis factor (TNF)-α, inhibit natural killer (NK) and CD8+ lymphocytes cytolytic activity, thus preventing antigen-presenting cells to be eliminated. The constant presence of antigen-presenting cells further enhances the inflammatory response driven by the IL-6 pathway, in particular [56]. Consequences of this inflammatory status are both direct and indirect: inflammation can directly promote tissue damage, as in the case of acute respiratory distress syndrome [57] and cardiovascular disease [58]. It is worth noting that the described mechanisms are almost identical to the ones witnessed in sepsis [59].

Cytokine storm, though, also promotes other infections: the constant inflammatory stimulus determines immune exhaustion, preventing an adequate immune response to any other stressor, including infections [60]. Interestingly, in this case, rather than proper sepsis, patients experience the persisting presence of the infectious agent, still burdened by a negative prognosis [61].

Studies carried out during the first stages of the pandemic highlighted the importance of IL-6 in triggering cytokine storm in SARS-CoV-2, thus monoclonal antibodies against this cytokine—that is, tocilizumab, sarilumab—were swiftly added to the tool bag of physicians fighting the disease [62]. Yet, results were mixed: while some centers reported very positive experiences with this class of drugs, even recommending supplementary doses if the first were not effective [63], some authors are far less enthusiastic [64].

Given the abnormal inflammatory response caused by SARS-CoV-2, patients suffering from this condition are exposed, in severe cases, to vascular manifestations secondary to thromboembolism and hypercoagulability, preferring lung tissue, unlike sepsis from other causes, which have a more rapid onset systemic organic involvement.

Overall, cytokine storm needs to be treated as fast as possible to try and reduce its negative effects, yet therapy timing can be tricky in the case of SARS-CoV-2 infection: blocking the inflammatory response too soon might block the body's response to SARS-CoV-2 while waiting too long might also prove useless, as inflammation might already be self-maintaining [65].

At the present moment, there is agreement on the lack of sufficient evidence in using this class of drugs in fighting against this disease [66].

Immune modulating drugs—that is, chloroquine and azithromycin—were also used, but their efficacy has not been consistent across different reports [67], [68], [69], [70], [71], [72]. One of the few immune-suppressing drugs approved in Covid are corticosteroids, particularly dexamethasone has proven effective in preventing the evolution of respiratory failure [73]. While consensus on their use in sepsis is still missing, it is worth noting that research suggests that corticosteroids could play a role in treating sepsis too [74]. However, all the associations between corticosteroid therapy, the severity and responsiveness of COVID-19 are still to be clarified, also because the individual variability of patients must always be taken into account. Another approach also includes the prevention of venous thromboembolism.

Overall, even though novel therapies are emerging, the most effective strategy in preventing severe SARS-CoV-2 infection is vaccination, which needs to be promoted as much as possible, particularly now, as there is growing anxiety about vaccinations [75,76].

## 4 Discussion

### 4.1 SARS-COV-2: promoting sepsis?

Many patients who have contracted SARS-CoV-2 infection have presented the diagnostic criteria for sepsis according to the International Third Consensus Definition of Sepsis [9]. In fact, this experimental study demonstrated the widespread localization of antiprocalcitonin antibodies affecting different structures of the organism, supporting the multisystem involvement which consequently leads to the death of the subject.

Sepsis is a very serious clinical syndrome that can be caused by the host's response to an infection by fungi, viruses, and in most cases by bacteria [77].

It has been estimated to affect about 49 million people every year, potentially contributing to up to 11 million deaths [78].

Some studies have compared the 2 conditions of sepsis-induced by SARS-CoV-2 and that from other causes. One study, for instance, compared sepsis with SARS-CoV-2 infection and found there are some similarities and differences [79]: both conditions can lead to acute respiratory failure and cytokine storm, abnormal coagulation, and in some cases disseminated intravascular coagulation, multiple organ dysfunction, elevated bilirubin, hypoxia, reduced glomerular filtration rate, hypoalbuminemia, and immunosuppression.

Yet, there also are some differences: venous thromboembolism and arterial thrombosis are much more frequent in SARS-CoV-2 infection which promotes thrombus formation locally, as opposed to sepsis which is associated with systemic hypercoagulation and reduced fibrinolysis. Also, while SARS-CoV-2 infection is a risk factor for the onset of sepsis, there is no evidence of the contrary. Important similarities were also found in the mortality in both conditions.

Several have tried to analyze both the similarities and differences between SARS-CoV-2-induced sepsis and that caused by other etiologic agents (Table 1).

**Table 1:** Characteristic of SARS-CoV-2-related sepsis and sepsis from other causes.

Characteristics of SARS-CoV-2-related sepsis and sepsis from other causes	
SARS-CoV-2-related sepsis	Venous thromboembolism and arterial thrombosis locally are much more frequent
	Risk factor for the onset of sepsis
General characteristics of sepsis	Endothelial dysfunction
	Immune dysregulation (cytokine storm)
	Hypercoagulability
	Acute respiratory failure
	Multiple organ dysfunction

In an interesting paper from 2020, Yataco et al. compare bacterial sepsis to SARS-CoV-2-related sepsis, highlighting that while for bacteria there are effective etiological therapies such as antibiotics, for SARS-CoV-2 at the moment only available some supportive therapies are available, such as venous thromboembolism prophylaxis, renal replacement therapy, and mechanical ventilation. The lethality rate in people on mechanical ventilation, that is, affected by the most severe form of SARS-CoV-2, is 88%, which is indeed very similar to that of patients who receive inappropriate antibiotic therapy, which stands at about 90% [80].

According to a Chinese study, in severe cases of SARS-CoV-2, lung infection stimulates alveolar macrophages and epithelial cells to synthesize proinflammatory cytokines and chemokines while at a systemic level the dysfunction of the microcirculation and cytokine storm cause viral sepsis, affecting other organs [81].

These hypotheses were confirmed by an Italian study that focused on the role of cytokine storm and endothelial dysfunction. Levels of cytokines and chemokines, including IL-6 and VEGF, are significantly increased in patients with SARS-CoV-2 infection. The cytokine storm stimulates the activity of monocytes, neutrophils, and macrophages which release a greater quantity of nitric oxide causing vasodilation [82].

One of the conditions that characterize SARS-CoV-2 sepsis is hypercoagulability. Several mechanisms can contribute to the explanation of this phenomenon. Systemic inflammation can activate the coagulation cascade resulting in activation of the fibrinolytic system, while another explanation is the direct attachment of the virus to Ace-2 endothelial cells.

Yet, while from a clinical point of view, the disturbances in coagulation that take place in sepsis and SARS-CoV-2 may appear similar, the disseminated intravascular coagulation that characterizes sepsis is different from that found in SARS-CoV-2 infection. In the latter there is a disturbance of the fibrinolytic system [83], while in the prior there is a diffuse consumption of all coagulation factors, leading to both ischemic and hemorrhagic events, in a condition known as disseminated intravascular coagulation (DIC) [84].

Another interesting aspect that needs to be taken into consideration is that, while viral sepsis is in itself a form of sepsis, SARS-CoV-2 increases the risk of developing both bacterial and fungal sepsis. In an Italian work, for instance, it was shown that patients admitted to intensive care units for SARS-CoV-2 infection had a twenty-fold greater risk of developing either a bacterial or a fungal bloodstream infection [85].

Once again, one of the causes seems to be the immune dysregulation caused by SARS-CoV-2 infection. Indeed, relative immune suppression can make vulnerable otherwise healthy patients to the development of sepsis. In numerous studies it has also been shown that there was a discrepancy between the lymphocyte count in patients who died from COVID-19, which was significantly decreased, and the blood levels of nonspecific markers of tissue damage, such as LDH, which were instead increased [86]. Also, it is worth noting that patients who develop sepsis during SARS-CoV-2 infections are those who are hospitalized, thus the hospitalization itself determines an increased risk of infection [87].

### 4.2 Histology results

**Fig. 3:**
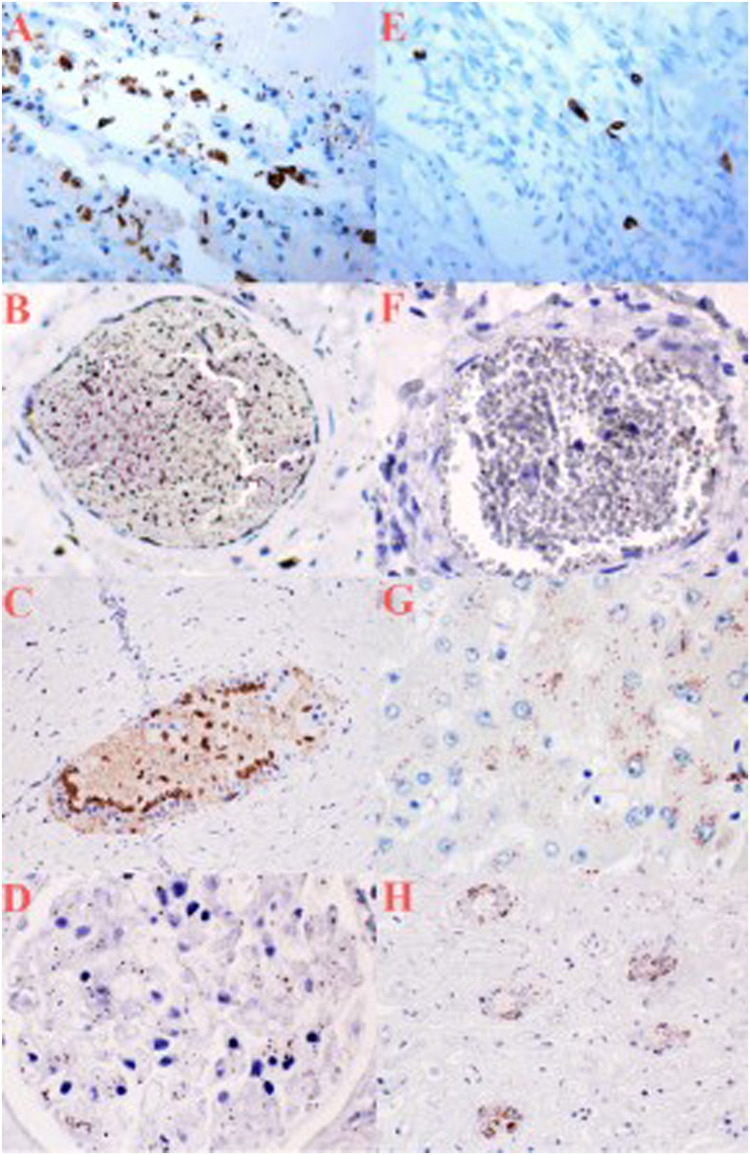
IHC behavior at antiprocalcitonin antibody. A (400×): lung cytoplasmic macrophages positivity; B–F (400×): blood vessels positivity; C (400×): ductal epithelium positivity; C (400×): ductal epithelium positivity; D (400×): glomerular positivity; E (400×): inflammatory cell positivity in lung alveolar septa; G (400×): hepatocyte positivity; H (400×): renal tubules positivity.

IHC results are summarized in Table 2 and exemplified in Fig. 3.

**Table 2:** IHC reaction evaluation according to the semiquantitative method selected, using an optic microscope and grading the positive reaction as follows: 0 (−) not expressed; 1 (+) isolated and disseminated expression; 2 (++) expression in scattered foci; 3 (+++) expression in widespread foci; 4 (++++) widespread expression.

Case	Lung	Liver	Kidney	Intravascular
Case 1	+	−	+	+
Case 2	+++	+	++	++
Case 3	−−	−	−	−
Case 4	++	−	+	+
Case 5	++	+	++	+++
Case 6	−	−	−	−
Case 7	++	−	+	++
Case 8	++++	++	+++	++++
Case 9	+	−	−	++
Case 10	−	−	−	−

A positive reaction was found concerning blood vessels in 7 cases; in the lung, clear staining in cytoplasm of myelomonocytic and inside the pneumocytes was noticed in 7 of 10 cases; IHC resulted positive in hepatocytes and in the ductal epithelium or in the portal-biliary space of the liver in 3 of 10 cases. In 6 of 10 kidney tissue samples, positive reaction was documented in the glomeruli and in the kidney tubules. The antiprocalcitonin antibody did not react in 3 cases of SARS-CoV-2-related deaths.

The antiprocalcitonin antibody exhibited no reaction in organs or blood vessels of cadavers who died from non-SARS-CoV-2 causes (control group).

These results suggest that there is a close connection between sepsis and Sars-cov-2 infection.

Ours is a preliminary study with important limitations, first and foremost the small number of cases selected. But in spite of this, we believe that our results can serve as a starting point for new and important research on this subject in the future.

## 5 Conclusions

While SARS-CoV-2 has completely reshaped health needs and resources, it did not magically eliminate other diseases. Chronic diseases have become an even heavier burden for patients [88], and medical emergencies have become even more challenging to deal with, because of the risk of infection for medical personnel [88,89].

Sepsis, in particular, presents several overlapping symptoms with Covid, thus it is sometimes difficult to identify the septic patient and start treatment fast enough. Also, sepsis can be both promoted and directly caused by SARS-CoV-2 infection, as shown by the experimental section of this manuscript, thus further complicating matters [90]. Treatment of SARS-CoV-2 septic shock is indeed burdened by even higher mortality than in sepsis caused by other agents [90] while treating sepsis in patients with SARS-CoV-2 infection might involve an immune system already shattered by a cytokine storm [91].

It is worth noting that the 2 diseases present a lot of similarities also in underlying pathogenetic mechanisms: the central role of IL-6 and TNF-α is common to the 2 and this is interesting from a therapeutic perspective. Yet, therapeutic options in sepsis heavily rely on antibiotic therapy, thus in the case of SARS-CoV-2. But even though support measures are the same, virus-targeted therapy is not available and probably not even efficient, as inflammation is self-maintained at this point.

Sepsis is a condition universally associated with worsening patient outcomes, as well as a significantly elevated mortality risk. In this historical context, where SARS-CoV-2 is also faced, whose full understanding and knowledge is still limited, it will be even more important to focus on prevention strategies, in order to limit the devastating effects that can be induced by this virus and its combination with bacterial infections.

Another fundamental aspect also concerns the scientific advances regarding the most suitable therapy in this type of patients, always taking into account the emerging problem of antibiotic resistance, especially in care-related infections that, in addition to increasing intrahospital mortality, increasingly generate medicolegal litigation.

Overall, it appears that these 2 disorders heavily interact with one another, clinically, but also in terms of burden, given the difficulty of diagnosing the 2 diseases, the potential they have to overlap, and the possible delays in therapy.
